# The association between C-reactive protein, mood disorder, and cognitive function in UK Biobank

**DOI:** 10.1192/j.eurpsy.2021.6

**Published:** 2021-02-01

**Authors:** David C. Milton, Joey Ward, Emilia Ward, Donald M. Lyall, Rona J. Strawbridge, Daniel J. Smith, Breda Cullen

**Affiliations:** 1 School of Medicine, Dentistry & Nursing, University of Glasgow, Glasgow, Scotland; 2 Institute of Health and Wellbeing, University of Glasgow, Glasgow, Scotland; 3 School of Life Sciences, University of Glasgow, Glasgow, Scotland; 4 Health Data Research UK, University of Glasgow, Glasgow, Scotland; 5 Cardiovascular Medicine Unit, Department of Medicine Solna, Karolinska Institutet, Stockholm, Sweden

**Keywords:** C-reactive protein, cognitive function, inflammation, mood disorder

## Abstract

**Background:**

Systemic inflammation has been linked with mood disorder and cognitive impairment. The extent of this relationship remains uncertain, with the effects of serum inflammatory biomarkers compared to genetic predisposition toward inflammation yet to be clearly established.

**Methods:**

We investigated the magnitude of associations between C-reactive protein (CRP) measures, lifetime history of bipolar disorder or major depression, and cognitive function (reaction time and visuospatial memory) in 84,268 UK Biobank participants. CRP was measured in serum and a polygenic risk score for CRP was calculated, based on a published genome-wide association study. Multiple regression models adjusted for sociodemographic and clinical confounders.

**Results:**

Increased serum CRP was significantly associated with mood disorder history (Kruskal–Wallis *H* = 196.06, *p* < 0.001, *η^2^* = 0.002) but increased polygenic risk for CRP was not (*F* = 0.668, *p* = 0.648, *η^2^* < 0.001). Compared to the lowest quintile, the highest serum CRP quintile was significantly associated with both negative and positive differences in cognitive performance (fully adjusted models: reaction time *B* = −0.030, 95% CI = −0.052, −0.008; visuospatial memory *B* = 0.066, 95% CI = 0.042, 0.089). More severe mood disorder categories were significantly associated with worse cognitive performance and this was not moderated by serum or genetic CRP level.

**Conclusions:**

In this large cohort study, we found that measured inflammation was associated with mood disorder history, but genetic predisposition to inflammation was not. The association between mood disorder and worse cognitive performance was very small and did not vary by CRP level. The inconsistent relationship between CRP measures and cognitive performance warrants further study.

## Introduction

Inflammation has for several years been linked with the development of mood disorder and cognitive impairment [1-3]. However, uncertainty remains regarding the extent of this relationship and the effects of serum inflammation levels compared to genetic predisposition toward inflammation.

C-reactive protein (CRP) is an inflammatory protein produced primarily by the liver as part of the acute phase response, which is initiated in response to changes in homeostasis due to insults such as infection or tissue injury. CRP levels rise exponentially as part of the acute phase response thus CRP is a useful biomarker for inflammation within the body. Once the responsible insult has been resolved, CRP levels rapidly decrease due to the protein having a short half-life in the blood. However, in chronic conditions, CRP levels can remain elevated to a lesser degree [4,5].

Elevated levels of CRP have been observed in people with bipolar disorder and have been associated with an increased risk of depression [6-8]. Increased CRP levels have also been shown to be associated with more severe symptoms of depression [9,10]. An association between increased CRP levels and worse cognitive function has been reported [11].

Evidence exists of a relationship between cognitive function and mood disorder with increased rates of cognitive impairment observed in those with depressive symptoms compared to those without [12,13]. Those with major depressive disorder and bipolar disorder have evidence of cognitive impairment during both euthymia and illness episodes compared to healthy individuals, suggesting an overlap between the mechanisms involved in mood disorders and cognitive impairment [14].

Inflammation may contribute to both mood disorder and cognitive impairment via several mechanisms. One of these is the cyclical theory of inflammation. Chronic inflammation can lead to the development of metabolic disease, resulting in even greater levels of inflammation within the body culminating in further disease development [1]. Similarly, patients with a history of repeated low-grade infections (evidence of a high inflammatory profile) are more likely to develop mood disorder in later life [15]. Other proposed mechanisms include dysregulation of the hypothalamic–pituitary–adrenal axis; cytokine-mediated neuronal destruction; altered concentrations of monoamine neurotransmitters; and decreased gray matter density in the brain [1,16-18].

Genome wide association studies (GWAS) have been used to investigate the specific common genetic variants associated with diseases or traits (single nucleotide polymorphisms; SNPs). Once SNPs associated with a trait are identified it is then possible to calculate a polygenic risk score (PRS), which represents an individual’s genetic predisposition for that trait based on the number of risk alleles they possess at those SNPs for the trait in question. PRS have been used to find evidence of genetic overlap between traits, including between inflammatory biomarkers and disease [19,20].

Although useful as a measure of an individual’s current inflammatory state, serum CRP levels can be influenced by confounding factors such as recent disease. PRSs have been used as a more stable reflection of lifetime potential for higher CRP. The use of a polygenic risk score for C-reactive protein level (PRS-CRP) within a study would enable assessment of an individual’s genetic predisposition toward inflammation excluding recent factors [21]. Including both serum CRP and PRS-CRP within the same study would ensure the effects of current and genetic levels of CRP were accounted for when investigating the relationship between CRP, mood disorder, and cognitive function.

To our knowledge, no study has investigated the association between serum CRP level, PRS-CRP, mood disorder, and cognitive function. This study may help to improve our understanding of the causes and consequences of mood disorder.

### Aims

The aim of this study of UK Biobank participants was to determine the magnitude of the associations between CRP, mood disorder, and cognitive function. In the first instance, the study aimed to assess whether serum CRP levels or PRS-CRP were increased in participants with a lifetime history of bipolar disorder, recurrent major depression, or a single episode of major depression. The study also aimed to investigate whether serum CRP levels were associated with PRS-CRP. Finally, the study aimed to determine whether an increased serum CRP level or PRS-CRP moderated the association between a lifetime history of mood disorder and cognitive performance.

## Methods

### Participants

This research has been conducted using the UK Biobank Resource under Application Number 11332 (PI: Cullen). Participants of the study were adults aged 40–70 who participated in the UK Biobank study between 2006 and 2010 and completed a baseline assessment at one of 22 assessment centers across the UK. The assessment process included a touchscreen questionnaire, a face-to-face interview with a research nurse, and measurement-taking during which participants were asked to give a blood sample. In the final 2 years of the recruitment period, a touchscreen questionnaire regarding mood disorder was added to the assessment process. Over 500,000 participants were recruited during this baseline phase of which 172,751 completed a touchscreen questionnaire regarding mood disorder [22,23]. The UK Biobank has approval from the NHS National Research Ethics Service as a research tissue bank (References 16/NW/0274 and 11/NW/0382).

Participants were excluded from the present study if they did not complete the mood disorder questionnaire; did not provide enough information to derive a mood disorder classification; or did not have data for serum CRP level or SNPs associated with CRP level. Participants were also excluded if they were not of white British ancestry; this was necessary because spurious associations arising from population stratification may occur if the study sample includes groups of individuals who differ systematically in both genetic ancestry and the phenotype of interest [24]. Also excluded were those who failed genotyping quality control and those who were closely related (third degree or closer) to other members of the cohort.

### History of mood disorder

Participants answered touchscreen questions relating to lifetime incidence of depressive and manic symptoms, and current mood state. A shortened and adapted version of the Patient Health Questionnaire formed the basis of questions asked on current depressive symptoms whilst the Structured Clinical Interview for DSM-IV Axis I Disorders was used to formulate questions on lifetime depressive and manic symptoms [25,26]. UK Biobank did not administer a measure of current manic symptom severity, due to time restrictions in the overall visit duration.

Participants were classified into different mood disorder groups based on their responses to the mood disorder questionnaire. The mood disorder groups were defined using criteria previously described by Smith et al. [27]. These criteria were based on self-reported lifetime experiences of features of BD or major depression. The groups included in the study were bipolar disorder type I (BD-I), bipolar disorder type II (BD-II), probable recurrent major depression (severe), probable recurrent major depression (moderate), and a single probable episode of major depression. A hierarchical structure was used during allocation of participants to one of the mood disorder groups. This hierarchy followed the order listed previously with BD-I deemed the most severe mood disorder and a single probable episode of major depression deemed the least severe. Participants who met the criteria for multiple groups were allocated to the most severe mood disorder for which they met the criteria. Participants who failed to meet the required criteria for classification into one of the defined mood disorder groups were included in a separate group defined as no mood disorder. This group acted as a control for the study. All groups were mutually exclusive. No structured questionnaires were administered by UK Biobank at this visit for other psychiatric conditions and it is possible that participants in any of the groups (including controls) had a history of other conditions apart from mood disorder.

### Cognitive function

To assess cognitive function participants were asked to complete several tasks on a touchscreen. These novel tasks were designed specifically for UK Biobank and completed by participants without supervision. Two tasks were administered throughout the baseline recruitment period and were analyzed in this study—reaction time and pairs matching (test of visuospatial memory). Both tasks have been shown to have moderate correlations with other standard cognitive tests in the same domains (*r* = 0.33–0.52), and both show moderate test–retest correlations over 4 weeks (*r* = 0.41–0.55) [28].

#### Reaction time

The participant was shown a pair of cards together on the touchscreen and asked to press a button if the cards shown were identical. In total, 12 pairs of cards were shown. Results were based on the mean time taken to press the button in milliseconds [29].

#### Pairs matching

The participant was shown multiple pairs of cards at once and asked to memorize the location of as many matching pairs as possible. The cards were turned face down and the participant was asked to touch as many pairs as possible in as few attempts as possible. Two rounds of matching were performed—first with three pairs of cards and then with six pairs of cards. Only the six-pair version was used in this study, to provide a sufficient range of data for analysis. Results were based on the number of errors made by the participant [30].

### Serum CRP level

Serum CRP level (mg/L) was measured by immunoturbidimetric high-sensitivity analysis on a Beckman Coulter AU5800 [31-33]. As this was not normally distributed, we report the median and quartiles below. Participants were also categorized into quintiles based on their serum CRP level with one representing the lowest serum CRP levels and five representing the highest serum CRP levels, to facilitate analysis of statistical interactions.

### **PRS** for CRP level

Genotyping, imputation, and quality control were done centrally by UK Biobank [34]. A PRS-CRP was calculated for each study participant based on 52 SNPs identified by Ligthart et al. in a GWAS meta-analysis as being significantly associated with serum CRP levels [35]. The PRS-CRP used in the study was created using 47 of these SNPs as data were not available, or did not have sufficient imputation quality, for the remaining five in the UK Biobank database. The score was an unweighted sum of the number of risk alleles each participant possessed across these 47 SNPs (missing SNPs for a given participant were coded as 0 unless all SNPs were missing, in which case the participant was excluded from analysis). To investigate whether this score should have been adjusted for genetic ancestry, the first 20 genetic principal components were included in a regression model. As this did not affect the results, the raw PRS-CRP score was used in the analyses reported in this study. However, batch was included as a covariate in multivariable regression models involving the PRS-CRP, to account for any confounding effects of the genotyping process.

### Covariates and background characteristics

Baseline sociodemographic, lifestyle, and clinical characteristics for each participant were obtained using the UK Biobank database. Potential covariates within the analyses were defined as age, sex, Townsend deprivation score (categorized into quintiles with one representing the least deprived and five representing the most deprived), educational status (dichotomized according to whether or not participants held a university/college degree), and current depressive symptoms (score ranging from zero to 12). Information on other background characteristics was also obtained consisting of smoking (“Never,” “Former,” “Current”), frequency of alcohol intake (“Daily or almost daily,” “3–4 times per week,” “1–2 times per week,” “1–3 times per month,” “Special occasions only,” “Never,”) body mass index (BMI; kg/m^2^), and current psychotropic medication use (list of medications reported by us previously) [36].

### Statistical analysis

The data were initially summarized using descriptive statistics. A Kruskal–Wallis H test assessed for differences in serum CRP level by mood disorder group, and a one-way analysis of variance (ANOVA) tested for group differences in PRS-CRP (as this was normally distributed). The relationship between serum CRP level and PRS-CRP was assessed with Spearman’s rank correlation coefficient. To enable comparison of the cognitive tests, the raw scores for each test were standardized to *z*-scores (mean = 0, standard deviation = 1) within 5-year age bands, with higher scores representing better performance. Two sets of multiple linear regression models were constructed to investigate associations between each measure of cognitive function (dependent variable), and mood disorder group and CRP (independent variables). One set of regression models included serum CRP level whilst the other included PRS-CRP. Each model accounted for confounders and tested for interactions between mood disorder group and the CRP variable included.

Statistical significance in the study was defined as *p* < 0.05. Correction for multiple comparisons was done where appropriate, for example, Bonferroni post-hoc pairwise comparisons following Kruskal–Wallis test. All analyses were conducted using IBM SPSS Statistics 26 except conversion of Kruskal–Wallis and ANOVA test statistics to effect size *η^2^*, which was done using the calculators provided at https://www.psychometrica.de/effect_size.html and https://osf.io/wgsi3/, respectively.

## Results

### Characteristics of the sample

**Figure 1.**
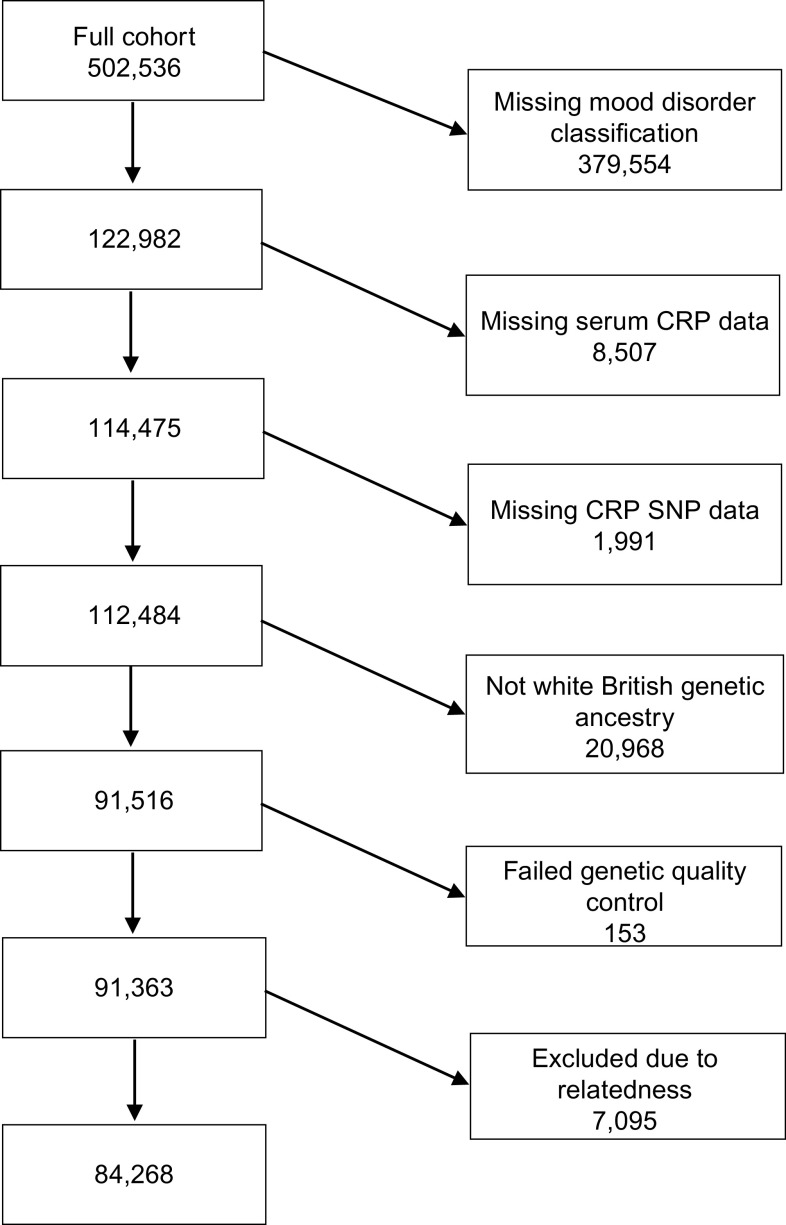
Flowchart showing the participants included in the study. CRP, C-reactive protein; SNP, single nucleotide polymorphism.

Of the 502,543 participants in the UK Biobank cohort, 84,268 were included in the study after the exclusion criteria had been applied (Figure 1).

Of the 84,268 study participants, 502 (0.6%) were classified into the BD-I group, 467 (0.6%) into the BD-II group, 5963 (7.1%) into the recurrent major depression (severe) group, 10,739 (12.7%) into the recurrent major depression (moderate) group, and 5739 (6.8%) into the single episode of major depression group. The remaining 60,858 (72.2%) were classified into the no mood disorder control group. The demographic and clinical characteristics of the participants are shown in Table 1. There was an over-representation of women in the depression groups, and of individuals in the most deprived Townsend deprivation score quintile in the BD-I group. The mean current depressive symptoms score was lowest in the control group with the highest witnessed in the BD-I group.

**Table 1.** Characteristics of the study participants at baseline.

	Bipolar disorder type I *n* = 502	Bipolar disorder type II *n* = 467	Recurrent major depression (severe) *n* = 5963	Recurrent major depression (moderate) *n* = 10739	Single episode major depression *n* = 5739	No mood disorder *n* = 60858
Age mean (SD), years	55 (8)	56 (8)	56 (8)	56 (8)	57 (8)	58 (8)
Gender female *n* (%)	233 (46.4)	208 (44.5)	3422 (57.4)	7321 (68.2)	3585 (62.5)	29657 (48.7)
Townsend score quintile						
Missing data *n*	0	1	11	25	11	78
1 (least deprived) *n* (%)	53 (10.6)	64 (13.7)	861 (14.5)	1861 (17.4)	1062 (18.5)	11803 (19.4)
2	66 (13.1)	85 (18.2)	1064 (17.9)	2213 (20.7)	1207 (21.1)	13771 (22.7)
3	100 (19.9)	80 (17.2)	1189 (20.0)	2263 (21.1)	1222 (21.3)	13400 (22.0)
4	130 (25.9)	120 (25.8)	1353 (22.7)	2377 (22.2)	1306 (22.8)	12586 (20.7)
5	153 (30.5)	117 (25.1)	1485 (24.9)	2000 (18.7)	931 (16.3)	9220 (15.2)
Educated to degree level						
Missing data *n*	2	1	29	46	22	452
Yes *n* (%)	171 (34.2)	169 (36.3)	2099 (35.4)	3608 (33.7)	2068 (36.2)	19377 (32.1)
Smoking status						
Missing data *n*	3	1	12	18	13	196
Current *n* (%)	102 (20.4)	94 (20.2)	937 (15.7)	1181 (11.0)	570 (10.0)	4790 (7.9)
Former	186 (37.3)	178 (38.2)	2264 (38.0)	3971 (37.0)	2128 (37.2)	21262 (35.0)
Never	211 (42.3)	194 (41.6)	2750 (46.2)	5569 (51.9)	3028 (52.9)	34610 (57.1)
Alcohol frequency						
Missing data *n*	1	0	3	2	4	17
Daily or almost daily *n* (%)	109 (21.8)	115 (24.6)	1240 (20.8)	2173 (20.2)	1180 (20.6)	13821 (22.7)
3–4 times per week	90 (25.2)	83 (17.8)	1182 (19.8))	2381 (22.2)	1398 (24.4)	15309 (25.2)
1–2 times per week	103 (20.6)	119 (25.5)	1322 (22.2)	2738 (25.5)	1480 (25.8)	16002 (26.3)
1–3 times per month	53 (10.6)	61 (13.1)	740 (12.4)	1415 (13.2)	700 (12.2)	6232 (10.2)
Special occasions only	76 (15.2)	38 (8.1)	812 (13.6)	1309 (12.2)	626 (10.9)	5939 (9.8)
Never	70 (14.0)	51 (10.9)	664 (11.1)	721 (6.7)	351 (6.1)	3538 (5.8)
Body mass index (kg/m^2^)						
Missing data *n*	3	2	36	47	23	225
Mean (SD)	28.4 (5.2)	27.9 (4.9)	28.0 (5.2)	27.8 (5.1)	27.5 (4.9)	27.2 (4.5)
On psychotropic medication						
Missing data *n*	9	7	70	156	95	765
Yes *n* (%)	258 (52.3)	65 (14.1)	2071 (35.1)	2023 (19.1)	470 (8.3)	2175 (3.6)
Current depressive symptoms score (0–12)						
Missing data *n*	30	38	418	753	385	4585
Mean (SD)	4 (3)	3 (3)	3 (3)	2 (2)	1 (2)	1 (1)
Serum CRP level (mg/L)						
Median (25th–75th percentile)	1.50 (0.73–3.43)	1.45 (0.68–3.02)	1.47 (0.71–3.03)	1.40 (0.68–2.90)	1.35 (0.68–2.73)	1.25 (0.63–2.56)
Serum CRP level quintile						
Missing data *n*	0	0	0	0	0	0
1 (lowest) *n* (%)	98 (19.5)	88 (18.8)	1098 (18.4)	2039 (19.0)	1102 (19.2)	12763 (21.0)
2	90 (17.9)	89 (19.1)	1109 (18.6)	2145 (20.0)	1193 (20.8)	13080 (21.5)
3	93 (18.5)	93 (19.9)	1180 (19.8)	2068 (19.3)	1148 (20.0)	12203 (20.1)
4	92 (18.3)	101 (21.6)	1249 (20.9)	2203 (20.5)	1141 (19.9)	11904 (19.6)
5	129 (25.7)	96 (20.6)	1327 (22.3)	2284 (21.3)	1155 (20.1)	10908 (17.9)
PRS for CRP level (0–94)						
Mean (SD)	54 (4)	54 (4)	55 (5)	54 (4)	54 (4)	54 (4)
Reaction time (ms)						
*z*-score^a^ mean (SD)	−0.09 (1.00)	−0.02 (0.96)	−0.08 (0.99)	−0.03 (0.96)	0.02 (0.96)	0.02 (0.97)
Pairs matching (errors)						
*z*-score^a^ mean (SD)	0.13 (1.05)	0.24 (1.00)	0.21 (1.06)	0.28 (1.03)	0.34 (1.02)	0.31 (1.03)

Abbreviations: CRP, C-reactive protein; mg/L, milligrams per liter; ms, milliseconds; PRS, polygenic risk score; SD, standard deviation.

a Higher *z*-score indicates better performance.

### Relationship between serum CRP level and mood disorder

The null hypothesis that serum CRP level was the same across all mood disorder groups was rejected (Kruskal–Wallis *H* statistic = 169.06, df = 5, *p* < 0.001, *η^2^* = 0.002). Post-hoc pairwise tests of differences in serum CRP level across the mood disorder groups are displayed in Table 2. A significant difference was observed between the no mood disorder group and the other mood disorder groups apart from the BD-II group (*p* = 0.480).

**Table 2.** Post-hoc pairwise tests of differences in serum CRP quintiles across mood disorder groups.

Mood disorder category	Mood disorder category	Test statistic	Adjusted *p*-Value
No mood disorder	Bipolar disorder type I	−3.257	0.017
	Bipolar disorder type II	−2.144	0.480
	Recurrent major depression (severe)	−9.428	<0.001
	Recurrent major depression (moderate)	−8.879	<0.001
	Single episode major depression	−4.608	<0.001
Bipolar disorder type I	No mood disorder	3.257	0.017
	Bipolar disorder type II	−0.721	1.000
	Recurrent major depression (severe)	−0.388	1.000
	Recurrent major depression (moderate)	−1.161	1.000
	Single episode major depression	−1.769	1.000
Bipolar disorder type II	No mood disorder	2.144	0.480
	Bipolar disorder type I	0.721	1.000
	Recurrent major depression (severe)	−0.589	1.000
	Recurrent major depression (moderate)	−0.141	1.000
	Single episode major depression	−0.748	1.000
Recurrent major depression (severe)	No mood disorder	9.428	<0.001
	Bipolar disorder type I	0.388	1.000
	Bipolar disorder type II	0.589	1.000
	Recurrent major depression (moderate)	−2.167	0.454
	Single episode major depression	−3.477	0.008
Recurrent major depression (moderate)	No mood disorder	8.879	<0.001
	Bipolar disorder type I	1.161	1.000
	Bipolar disorder type II	0.141	1.000
	Recurrent major depression (severe)	2.167	0.454
	Single episode major depression	−1.792	1.000
Single episode major depression	No mood disorder	4.608	<0.001
	Bipolar disorder type I	1.769	1.000
	Bipolar disorder type II	0.748	1.000
	Recurrent major depression (severe)	3.477	0.008
	Recurrent major depression (moderate)	1.792	1.000

### Relationship between PRS for CRP level and mood disorder

One-way ANOVA indicated no significant difference between the mood disorder groups in terms of PRS-CRP (*F*(5, 84262) = 0.668, *p* = 0.648, *η^2^* = 0.002).

### Relationship between serum CRP level and PRS for CRP level

The Spearman’s rank correlation coefficient was 0.167 (*p* < 0.001) showing significant evidence of a small positive correlation between serum CRP level and PRS-CRP.

### Relationship between cognitive function, mood disorder, and serum CRP level

Separate regression models were conducted for the dependent variables of reaction time (Table 3) and pairs matching (Table 4), with the same independent variables and covariates.

**Table 3.** Multiple regression results for the relationship between reaction time *z*-score, mood disorder group and serum CRP level quintile before and after adjusting for confounders.

	Unadjusted model		Fully adjusted model^a^	
	Coefficient (95% CI)	*p-*Value	Coefficient (95% CI)	*p*-Value
Reaction time *z*-score^b^				
Serum CRP level quintile				
2	0.002 (−0.018 to 0.023)	0.830	0.007 (−0.014 to 0.028)	0.512
3	−0.019 (−0.040 to 0.001)	0.067	0.001 (−0.021 to 0.022)	0.963
4	−0.030 (−0.051 to −0.009)	0.004	0.004 (−0.017 to 0.026)	0.696
5	−0.089 (−0.110 to −0.068)	<0.001	−0.030 (−0.052 to −0.008)	0.007
Bipolar disorder type I	−0.111 (−0.196 to −0.025)	0.011	−0.064 (−0.152 to 0.024)	0.156
Bipolar disorder type II	−0.042 (−0.130 to 0.046)	0.354	−0.032 (−0.123 to 0.060)	0.495
Recurrent major depression (severe)	−0.100 (−0.126 to −0.074)	<0.001	−0.058 (−0.085 to −0.030)	<0.001
Recurrent major depression (moderate)	−0.049 (−0.069 to −0.029)	<0.001	−0.002 (−0.024 to 0.019)	0.829
Single episode major depression	−0.003 (−0.029 to 0.023)	0.809	0.016 (−0.011 to 0.043)	0.236

Abbreviations: CI, confidence interval; CRP, C-reactive protein.

a Full model adjusted for age, gender, Townsend deprivation score, educational status, current depressive symptoms.

b Linear regression; omitted reference group was the mood disorder control group and serum CRP quintile 1; coefficients are in *z*-score units (higher = better performance).

**Table 4.** Multiple regression results for the relationship between pairs matching *z*-score, mood disorder group and serum CRP level quintile before and after adjusting for confounders.

	Unadjusted model		Fully adjusted model^a^	
	Coefficient (95% CI)	*p-*Value	Coefficient (95% CI)	*p-*Value
Pairs matching *z*-score^b^				
Serum CRP level quintile				
2	0.021 (0.000 to 0.043)	0.055	0.032 (0.010 to 0.055)	0.005
3	0.030 (0.008 to 0.052)	0.008	0.054 (0.031 to 0.077)	<0.001
4	0.029 (0.007 to 0.051)	0.010	0.060 (0.037 to 0.083)	<0.001
5	0.027 (0.005 to 0.049)	0.019	0.066 (0.042 to 0.089)	<0.001
Bipolar disorder type I	−0.180 (−0.272 to −0.088)	<0.001	−0.163 (−0.258 to −0.067)	0.001
Bipolar disorder type II	−0.074 (−0.169 to 0.022)	0.130	−0.065 (−0.164 to 0.034)	0.200
Recurrent major depression (severe)	−0.104 (−0.131 to −0.076)	<0.001	−0.095 (−0.125 to −0.065)	<0.001
Recurrent major depression (moderate)	−0.029 (−0.050 to −0.007)	0.009	−0.025 (−0.048 to −0.002)	0.036
Single episode major depression	0.032 (0.004 to 0.060)	0.026	0.027 (−0.002 to 0.056)	0.070

Abbreviations: CI, confidence interval; CRP, C-reactive protein.

a Full model adjusted for age, gender, Townsend deprivation score, educational status, current depressive symptoms.

b Linear regression; omitted reference group was the mood disorder control group and serum CRP quintile 1; coefficients are in *z*-score units (higher = better performance).

The unadjusted model for reaction time *z*-score showed serum CRP level quintiles four and five, BD-I, recurrent major depression (severe), and recurrent major depression (moderate) were significantly associated with lower cognitive performance. In the adjusted model for reaction time, only serum CRP level quintile five and recurrent major depression (severe) remained significantly associated with lower cognitive performance. As mood disorder severity increased larger decreases in performance were observed—with the exception that recurrent major depression (severe) showed a larger decrease in performance compared to BD-II. Most of the effect sizes decreased with adjustment for confounders.

The unadjusted pairs matching model showed serum CRP quintile three, four, and five, and a single episode of major depression were significantly associated with higher cognitive performance whilst BD-I, recurrent major depression (severe), and recurrent major depression (moderate) were significantly associated with lower cognitive performance. In the adjusted model all serum CRP quintiles were significantly associated with a higher pairs matching *z*-score whilst BD-I, recurrent major depression (severe), and recurrent major depression (moderate) all remained significantly associated with a lower pairs matching *z*-score. The favorable effect sizes increased in line with increasing serum CRP level quintiles. Compared to the unadjusted model, the effect sizes increased for all serum CRP quintiles whilst they decreased for all mood disorder groups.

### Relationship between cognitive function, mood disorder, and PRS for CRP level

The results of these models are shown in Table 5. The unadjusted models showed PRS-CRP was not significantly associated with either reaction time *z*-score or pairs matching *z*-score. There were significant differences by mood disorder group, however: BD-I was associated with worse performance on both cognitive tests, and a single episode of major depression was associated with a higher pairs matching *z*-score. After adjusting for confounders, PRS-CRP remained unassociated with reaction time *z*-score but became significantly associated with a higher pairs matching *z*-score.

**Table 5.** Multiple regression results for the relationship between cognitive function, mood disorder group, and PRS for CRP level before and after adjusting for confounders.

	Unadjusted model		Fully adjusted model^a^	
	Coefficient (95% CI)	*p-*Value	Coefficient (95% CI)	*p*-Value
Reaction time *z*-score^b^				
PRS for CRP level	0.000 (−0.001 to 0.002)	0.942	0.000 (−0.002 to 0.002)	0.967
Bipolar disorder type I	−0.116 (−0.202 to −0.031)	0.008	−0.064 (−0.154 to 0.024)	0.152
Bipolar disorder type II	−0.045 (−0.133 to 0.043)	0.318	−0.033 (−0.125 to 0.058)	0.477
Recurrent major depression (severe)	−0.104 (−0.130 to −0.078)	<0.001	−0.059 (−0.087 to −0.032)	<0.001
Recurrent major depression (moderate)	−0.052 (−0.072 to −0.032)	<0.001	−0.003 (−0.024 to 0.019)	0.799
Single episode major depression	−0.005 (−0.032 to 0.021)	0.688	0.015 (−0.012 to 0.042)	0.273
Pairs matching *z*-score^b^				
PRS for CRP level	0.001 (0.000 to 0.003)	0.134	0.002 (0.000 to 0.004)	0.011
Bipolar disorder type I	−0.179 (−0.271 to −0.087)	<0.001	−0.162 (−0.258 to −0.066)	0.001
Bipolar disorder type II	−0.073 (−0.168 to 0.022)	0.132	−0.063 (−0.162 to 0.037)	0.216
Recurrent major depression (severe)	−0.103 (−0.131 to −0.075)	<0.001	−0.093 (−0.123 to −0.063)	<0.001
Recurrent major depression (moderate)	−0.028 (−0.049 to −0.007)	0.010	−0.023 (−0.046 to −0.000)	0.049
Single episode major depression	0.032 (0.004 to 0.061)	0.023	0.029 (0.000 to 0.058)	0.052

Abbreviations: CI, confidence interval; CRP, C-reactive protein; PRS, polygenic risk score.

a Full model adjusted for age, gender, Townsend deprivation score, educational status, current depressive symptoms, genotyping batch.

b Linear regression; omitted reference group was the mood disorder control group; coefficients are in *z*-score units (higher = better performance).

The adjustment for confounders resulted in a decrease in the magnitude of cognitive differences associated with mood disorder in almost every group, with some results no longer significant. Recurrent major depression (severe) was significantly associated with a lower reaction time *z*-score and pairs matching *z*-score whilst BD-I and recurrent major depression (moderate) were significantly associated with a lower pairs matching *z*-score only. The adjusted effect sizes across mood disorder groups were generally larger for pairs matching compared to reaction time, although all effect sizes were very small in absolute terms.

### Interactions between CRP level and mood disorder

Tests for interactions between serum CRP level and mood disorder, and PRS-CRP and mood disorder, were conducted in the adjusted models for each cognitive test. One significant interaction was observed in one of the pairs matching models (between serum CRP level quintile three and recurrent major depression (severe)), but in the context of otherwise non-significant interaction coefficients this was thought to be a spurious finding and so stratified models were not conducted.

## Discussion

In this large cohort of individuals of white British ancestry in middle to early old age, some evidence was found of associations between CRP, mood disorder, and cognitive function. Our results suggested that increased serum CRP level was significantly associated with a history of mood disorder whereas an increased PRS-CRP was not. However, a significant (albeit small) positive correlation was observed between serum CRP level and PRS-CRP. We found, after adjusting for confounders, that the highest serum CRP levels were significantly associated with worse performance on a reaction time test. Conversely, and unexpectedly, higher serum and PRS-CRP levels were significantly associated with better performance on the pairs matching visuospatial memory test. Importantly, these findings may not be of clinical significance as the coefficients for the differences in cognitive performance observed—whether higher or lower—were very small.

The observed association between mood disorder and worse cognitive function is in line with previous research [12-14]. Significantly lower reaction time performance was observed in the recurrent major depression (severe) group, whilst BD-I, recurrent major depression (severe), and recurrent major depression (moderate) were significantly associated with lower pairs matching performance (this was borderline in the case of recurrent major depression (moderate)). As mood disorder increased in severity, the negative association with cognitive function tended to increase in magnitude, with BD-I associated with the largest difference in cognitive function. Again the coefficients for these differences were very small so these associations may not be clinically significant although further research may be warranted to determine this. The overall absence of significant interactions between CRP measures and the mood disorder groups suggests that the relationship between mood disorder history and cognitive function did not vary substantially across different levels of CRP.

It was reasonable to hypothesize that increased levels of inflammation would be associated with mood disorder history and a reduction in cognitive performance as shown by previous studies [6,7,11]. As such, the mixed results in our study are surprising. The lack of significant association between PRS-CRP and mood disorder was perhaps unexpected although the positive correlation between serum CRP level and PRS-CRP shows there may be more to investigate regarding this relationship. It is unclear why increased CRP levels were associated with both positive and negative effects on cognitive performance.

This study is one of the first to explore the association between CRP and mood disorder incorporating both serum CRP level and PRS-CRP. Many studies have investigated the association between serum CRP level and psychiatric disorder, but few have also included PRS-CRP [8,37,38]. One previous study investigated the relationship between PRS-CRP and various psychiatric and somatic disorders but used a PRS comprising of only 18 SNPs from an older GWAS [39]. We have extended this area of research by comparing serum CRP levels, using a PRS-CRP including 47 SNPs, and investigating psychiatric disorders as well as cognition.

The inclusion of serum and genetic CRP variables within the same study allowed us to compare the effect of current measured inflammation levels, versus genetic predisposition to inflammation, on mood disorder history and cognitive performance. The use of a 47 SNP PRS-CRP represents an advance on previous research, but there is scope for a future study to use the full GWAS results from the Ligthart meta-analysis to construct a weighted PRS across all the CRP-associated SNPs identified, rather than just the genome-wide significant SNPs used in this study [35].

### Limitations

The UK Biobank cohort is not representative of the UK population. Participants are more likely to live in less socio-economically deprived areas, be more highly educated and report fewer health conditions than the general population [40]. We also restricted our analyses to white British participants. The results of this study are therefore not generalizable to the UK population. In particular, the time and effort required to opt into participate and attend a detailed assessment visit may have meant that people with worse cognitive function and/or more severe affective symptoms are under-represented in the UK Biobank cohort. It is also likely that the UK Biobank cohort is less socio-economically deprived and more healthy than clinical samples used in some previous studies of CRP and mental health. The restricted age range of the cohort may be a further limitation and it will be informative to investigate age-related differences in the associations of interest as the cohort is followed up longitudinally.

This study did not investigate the relationship between cardiometabolic health and CRP levels. Increased BMI has been shown to be associated with higher serum CRP levels whilst increased CRP levels have been found to be associated with medical conditions such as hypertension and type 2 diabetes [41,42]. Accounting for BMI and conditions associated with increased CRP levels may improve our understanding of the results obtained from this study. Future research should approach these questions using a mediation or structural equation modeling framework to map out the assumed pathways between mood disorder, inflammation, and cognitive function, to understand the potential mediating role of cardiometabolic disease and associated risk factors including smoking, alcohol, and obesity.

Another limitation of the study was that CRP was the only inflammatory biomarker studied. Many inflammatory biomarkers such as interleukin-1 and tumor necrosis factor alpha are thought to be associated with mood disorder and cognitive impairment [43,44]. Investigating these other biomarkers may better explain the association between inflammation, mood disorder, and cognitive function, as they reflect more specific inflammatory pathways rather than the general inflammatory levels which are indicated by CRP. Lastly, we note that only two cognitive tests were analyzed in this study; future studies should make use of additional cognitive measures administered at follow-up waves, to investigate potential group differences in other cognitive domains such as verbal memory and executive function.

## Conclusion

Our findings extend the work previously done in this area and indicate that current measured inflammation levels appear to be of greater relevance than lifetime genetic tendency toward inflammation, with regard to mood disorder history. It may be that inflammatory events during life have a larger bearing on the development of mood disorder compared to genetic predisposition toward increased levels of inflammation. However, there was a significant positive correlation between serum CRP level and genetic predisposition to higher CRP, which may mean that a relationship does exist between PRS-CRP and mood disorder but could not be detected with the available data in this study. The association between mood disorder and lower cognitive performance was small and did not appear to be moderated by CRP level. The finding that CRP measures were significantly associated with both positive and negative differences in cognitive performance requires further investigation.

## Data Availability

The first author and corresponding author had full access to the study data. UK Biobank is an open-access resource. Data are available to bona fide scientists, undertaking health-related research that is in the public good. Access procedures are described at http://www.ukbiobank.ac.uk/using-the-resource/. Statistical analysis code for this study will be publicly available at the corresponding author’s OSF project page: https://osf.io/tngqh/.
